# Effects of Helminth Eradication on the Immune System

**DOI:** 10.20411/pai.v2i2.205

**Published:** 2017-07-10

**Authors:** Ziva Weisman, Alexander Kalinkovich, Miguel Stein, Zalman Greenberg, Gad Borkow, David Adlerstein, Jemal Ali Mahdi, Zvi Bentwich

**Affiliations:** 1 Kaplan Medical Center, Ben-Ari Institute of Clinical Immunology and AIDS Center, Rehovot, Israel; 2 Hebrew University Hadassah Medical School, Jerusalem, Israel; 3 Public Health Laboratory, Ministry of Health, Jerusalem, Israel; 4 Department of Microbiology, Immunology and Parasitology, College of Medicine and Health Sciences, University of Gondar, Gondar, Ethiopia; 5 Department of Microbiology Immunology and Genetics, Center for Emerging and Tropical Diseases and AIDS, Ben Gurion University of the Negev, Beer Sheba, Israel

**Keywords:** Helminth infections, helminths immunology, antihelminth treatment

## Abstract

**Introduction:**

Helminth infection has a profound effect on the immune system. However, the precise nature of the immune changes that are elicited by helminth infection have not been sufficiently characterized. Furthermore, the reversibility of these changes after treatment has not been documented sufficiently. We studied the immune profiles of Ethiopian immigrants to Israel at baseline, that is on arrival and at one-year follow-up and compared individuals who received antihelminth treatment during the study period with those who missed the treatment.

**Methods:**

A longitudinal follow up study involving different groups of subjects was conducted. Baseline data was recorded from the newly arrived Ethiopian immigrants for a series of peripheral blood tests, including: IgE and Eosinophil levels, T-cell populations, T-cell receptor phenotypes, and cytokine measurement. These tests were all repeated after a 1-year interval. Results were compared between the newly arrived Ethiopian immigrants (NEW-Eth-Il), long term Ethiopian immigrants (LT-Eth-Il), and non Ethiopian Israeli controls (NON-Imm-Il).

**Results:**

Of the 184 individuals, 111 were NEW-Eth-Il, who had a high prevalence of helminth infection, the immunological changes were elevated IgE levels and eosinophil counts, decreased CD4/CD8 ratio, increased proportion of HLA-DR+CD3+, HLA-DR+CD4+ and HLA-DR+CD8+ cells, decreased proportion of CD45RA+CD4+ (naive) and CD28+CD8+ cells, increased proportion of CD45RO+CD4+ (memory) cells, and increased secretion of IL-4 and IL-5 (Th2 type cytokines). In the 42 LT-Eth-Il participants, who all had negative tests for helminth infection, we did not observe these immune changes and their immune profile did not differ markedly from that of the NON-Imm-Il controls. The follow-up immune profiles of 33 NEW-Eth-Il who received succesful antihelminth treatment, showed a significant normalization in the above-mentioned variables that was not observed in the 19 NEW-Eth-Il who missed and did not receive the antihelminth treatment.

**Conclusions:**

These findings demonstrate that helminth infection is associated with profound immune changes that are normalized within a short time after helminth eradication. They also strengthen the hypothesis that effective antihelminth interventions, in areas endemic for intestinal helminths, may have an impact on AIDS and tuberculosis epidemics.

## INTRODUCTION

Helminth infections affect over 1.5 billion people worldwide, mostly in developing countries [1–3]. These chronic infections, are acquired in early childhood and commonly persist into old age if left untreated. In addition to well-known morbidities such as malnutrition, anemia, and impaired physical and cognitive development, helminth infection also causes significant changes to the host immune system.

Those changes that have been well-described, by ourselves and others, include eosinophilia, elevated serum IgE, a Th2-dominant cytokine profile (increased IL-4, IL-5, and IL-10, decreased IL-2 and IFNγ [4–8], decreased levels of CD4+ lymphocytes and a decreased CD4 to CD8 ratio, increased immune activation and apoptosis [4, 9, 10], impaired immune response to recall antigens [11, 12], impaired signal transduction, and anergy [13, 14]. Some of these changes are reversible with antihelminth treatment [15]. However, the rate of normalization varies and depends on additional factors such as helminth burden [16, 17].

We have previously hypothesized that helminth infection and its associated immune abnormalities are a major contributor to the rapid spread and progression of HIV infection in developing countries [18–20]. Such changes may increase the risk for HIV infection and cause a more rapid progression to AIDS [18, 16, 21, 22]. Thus, the implementation of effective deworming programs in Africa and throughout the developing world may be critical in the fight against HIV/AIDS and other infectious diseases, such as tuberculosis [23–25].

In the last few decades a large immigration of Ethiopian Jews, with and without HIV infection, arrived in Israel. While providing medical care for this population, we observed that a high percentage were infected by helminths [26]. We also observed profound immune abnormalities characterized by a widespread immune activation and dysregulation with a dominant Th2 type of immune response [9, 20]. After treatment of their underlying helminth infection, we observed a significant normalization of immune profile, including a decrease in eosinophilia and T-cell activation, after only 6 to 2 months. These findings and their possible relevance to the AIDS and tuberculosis epidemics in the developing world, have prompted this report.

## MATERIALS AND METHODS

### Study design and study population

A longitudinal follow-up study was conducted in a total of 184 individuals: 153 were Ethiopian immigrants and 31 were non-Ethiopian Israelis (*NON-Imm-Il*). Of the 153 Ethiopian immigrants, 111 had arrived in Israel within the previous 1 to 3 months (*NEW-Eth-Il*) and 42 had resided in Israel for over 5 years (*LT-Eth-Il*). All subjects had negative test results for HIV-1, HIV-2, tuberculosis, malaria, hepatitis, and syphilis.

Ethiopian immigrants to Israel usually have a complete clinical check up including stool examination for worm infections on their arrival. This was accompanied by a deworming program and follow-up during the ensuing months. Regretfully, some individuals from this group missed the baseline deworming and the 6-month follow-up and thereby formed a special group. They were eventually treated at the end of the first year after their arrival in Israel. This unplanned situation allowed us to form 2 groups: a treated group versus an untreated group and to compare them with the other groups, that is those immigrants that had arrived in Israel over 5 years previously and the non-Ethiopian Israeli controls.

We studied the effect of helminth eradication on the immune system by measuring several immunological markers in the 2 subgroups of the *NEW-Eth-Il*: Group-A, 19 individuals who were chronically infected and living in the same environment, but who did not receive antihelminth treatment and remained infected with helminths during the whole study period, and Group-B, 33 individuals who were chronically infected with 1 or more helminth species but underwent treatment. It was therefore possible to compare their immune profiles at baseline with those at 6 to 12 months after succesful antihelminth treatment.

### Stool Analysis

Stool samples were collected from the *NEW-Eth-Il* group within 6 months of their arrival and from the *LT-Eth-Il* on enrollment in the study. These samples were stored at 4°C until examined. The presence and amount of parasite eggs in the stool specimens were determined by a formol-ether sedimentation method [27]. Infected persons received the antihelminth drugs albendazole and/or praziquantel within 6 to 12 months of their arrival in Israel. Albendazole (400 mg/ day) was given for 3 consecutive days and the dosage repeated after a week. Praziquantel was given in a single dose of 40 mg/kg. Then second stool samples were taken and examined for the presence of eggs 3 to 6 months after treatment. If these samples remained positive, treatment was repeated.

### Blood examinations

Blood samples were collected from the *NEW-Eth-Il* group before treatment and 6 to 12 months later, and from the *LT-Eth-Il* and *NON-Imm-Il* groups only once on their visit to the clinic. Plasma samples were kept frozen at -20°C until tested. Blood cell counts and differentiation were routinely measured in the Hematology Department of the Kaplan Medical Center. Plasma IgE levels were determined by Delfia total IgE Fluoroimmunoassay Total IgE Kit (Wallac Oy, Turku, Finland) according to the manufacturer's instructions.

### Lymphocyte phenotype analysis

Flow cytometric measurements were made on whole blood using a FACScan (Becton Dickinson Immunocytometry System, San Jose, CA) within 6 hours after blood collection into EDTA-containing tubes as previously described in detail (9). Fluorescein isothiocyanate (FITC) or phycoerythrin (PE) labeled antibodies to CD3, CD4, CD8, CD28/CD8 (Becton Dickinson), HLADR/CD3, HLA-DR/CD4, HLA-DR/CD8, CD45RA/CD4, CD45RA/CD8, CD45RO/CD4, and CD45RO/CD8 (Dako, Glostrup, Denmark) were used. Cells incubated with FITC- or PE-conjugated mouse IgG1/IgG2a (Dako) served as the isotype control. Lymphocytes were distinguished from monocytes on the basis of their forward versus side scatter pattern. A minimum of 10,000 cells per sample was analyzed by CELLQuest software (Becton Dickinson).

### Cytokine secretion

Peripheral blood mononuclear cells (PBMC) were obtained from heparinized venous blood by standard centrifugation over Histopaque (Sigma, Nes-Ziona, Israel). Cells were washed and resus-pended at 2x10^6^ cells/ml in RPMI (Biological Industries Co, Beit-Haemek, Israel) supplemented with 5% heat inactivated pooled human AB serum (Sigma), 2mM L-glutamine and 1% penicillin, streptomycin, and nystatin (Biological Industries). Cells (1 ml/well) were cultured for 72 hours in 24-well multidish plates (Nunc A/S, Roskilde, Denmark) at 37°C under 6.5% CO_2_ with or without phytohaemagglutinin (PHA; 1:100, Difco PHA-P; Detroit, MI). Supernatants were collected by centrifugation and stored at -70°C until tested.

Levels of IFN-γ and IL-4 in the supernatants were determined by Cytoscreen (BioSource International Inc., Camarillo, CA) and Endogen (Endogen Inc., Woburn, MA) ELISA kits; IL-5 levels were measured by Biotrak (Amersham Pharmacia Biotech UK Ltd, Little Chalfont, Buckingham-shire, England) ELISA kit. Cytokine measurements presented for all cytokines are after subtraction of cytokine concentrations in supernatants from unstimulated cultures from concentrations in supernatants from stimulated cells.

### Statistical analysis

Statistical analysis was performed with SigmaStat software for Windows Version 2 (Jandel Corporation^R^, Chicago, IL). Student's *t* -test and paired *t* -test were used for comparing the means of different groups and between 2 consecutive tests for each individual, respectively. For non-parametric data, the Mann Whitney rank test and Wilcoxon signed rank test were used. Values of *P* < 0.05 was considered significant.

### Ethical clearance

The study was conducted on subjects visiting for clinical follow-up. Institutional approval was received to collect the data, and the data were analyzed anonymously. All participants with helminth infections were treated as per the protocol of the clinic.

## RESULTS

Baseline stool analysis showed that participants in the *LT-Eth-Il* and *NON-Imm-Il* groups had negative results for helminth infection. The *NEW-Eth-Il* (groups A and B) were found to be highly infected with helminth parasites irrespective of age or sex (Table 1). Seventy-three percent were infected by at least one parasite species and of those, 34% were infected by 2 or more parasite species, with no significant differences between group A and B (Table 1). The prevalence of specific species were: *Necator americanus* (36.8%), *Ascaris lumbricoides* (29.4%), *Schistosoma mansoni* (26.5%), *Trichuris trichiura* (24.2%), *Taenia saginata* (8.2%), and *Hymenolepis nana* (3.6%). No notable reduction in the egg loads was found in individuals that were not treated for helminthiasis, with the exception of *A. lumbricoides,* which almost disappeared within a year after immigration.

**Table 1.** Parasite distribution and intensity of infection in Group A (in which helminths were not eradicated) and Group B (in which helminths were eradicated).

Parasite	Group A (n = 19)		Group B (n = 33)	
	Infected subjects n (%)	Eggs/ml (mean)	Infected subjects n (%)	Eggs/ml (mean)
*Necator americanus*	9 (47.3)	117	22 (66.7)	108
*Schistosoma mansoni*	16 (84.2)	81	18 (54.5)	30
*Ascaris lumbricoides*	10 (52.6)	754	17(51.5)	793
*Trichuris trichiura*	4 (21)	18	7 (21.2)	14
*Taenia saginata*	2 (10.5)	270	2 (6.1)	25
*Hymenolepis nana*	1 (5.2)	10000	2 (6.1)	5009
2 or more parasites	13 (68.4)	NA^§^	21 (63.6)	NA

^§^NA: Not Applicable

### Immune profile of the studied groups

We compared the immune profiles of the 3 subgroups. The 111 *NEW-Eth-Il* participants were highly infested with helminths at baseline. Their immune profiles were significantly different than that of the *NON-Imm-Il* group indicated by marked eosinophilia and increased IgE levels in peripheral blood, a significant decrease in CD4/CD8 ratio, increased percentage of activated (HLA-DR+) cells in all T-lymphocyte subsets (CD3+, CD4+ and CD8+ cells), decreased percentage of CD45RA+CD4+ cells and CD28+CD8+ cells, and increased percentage of memory CD45RO+CD4+ cells (Table 2).

**Table 2.** Immunological parameters of study groups at baseline: new and long term-Ethiopian immigrants and non-Ethiopian Israeli residents.

Parameters	*NEW-Eth-Il* (n = 111)	*LT-Eth-Il* (n = 42)	*NON-Imm-Il* (n = 31)
Eosinophils (cells/μl)	748 (617) ***	322 (282)***^§§§^	127 (47)
IgE (IU/ml)	1650 (1316)***	203 (218)**^§§§^	105 (130)
CD3 (%)	73.5 (8.1)	74.2 (6.3)	72.7 (5.5)
CD4 (%)	36.4 (7)***	44.2 (15.8)^§§§^	41.3 (5.7)
CD8 (%)	37.2 (9.4)***	33.4 (14.6)	30.7 (4.6)
CD4/CD8 (ratio)	1.06 (0.41)***	1.38 (0.39)^§§§^	1.37 (0.37)
HLA-DR+CD3+ (%)	10.3 (7.9)***	5.9 (3.8)^§§^	4.9 (2.2)
HLA-DR+CD4+ (%)	6.3 (3.6)***	4.2 (2.5)*^§§^	3 (1.1)
HLA-DR+CD8+ (%)	13.4 (8.9)***	9 (14.6)***	5.9 (2)
CD45RA+CD4+ (%)	24.2 (9.5)***	32.2 (9.4)^§§§^	35.3 (12.3)
CD45RA+CD8+ (%)	61.5 (14.6)	69.8 (9.4)^§§^	65.8 (13.7)
CD45RO+CD4+ (%)	68.3 (11.5)***	65.2 (10.5)***	58.2 (8.6)
CD45RO+CD8+ (%)	31.1 (16)	29.0 (9.6)	34.1 (10.6)
CD28+CD8+ (%)	40.2 (13.3)***	51.3 (13.3)^§§§^	55.7 (15.6)

Results are presented as means ± SD

*Significantly different from *NON-Imm-Il* group, *P < 0.05, **P < 0.01, ***P < 0.001

^§§^Significantly different from *NEW-Eth-Il* group, ^§§^*P* < 0.01, §§§*P* < 0.001

In contrast, the immune profile of the *LT-Eth-Il* group resembled that of the *NON-Imm-Il*, with the following exemptions: elevated levels of blood eosinophils and IgE and percentage of HLADR+CD4+, HLA-DR+CD8+ and CD45RO+CD4+ cells. In general, the immune profile of the *LT-Eth-Il* was significantly less activated than that of the *NEW-Eth-Il* group (Table 2).

### Normalization of the immune profile after successful antihelminth treatment

**Figure 1.**
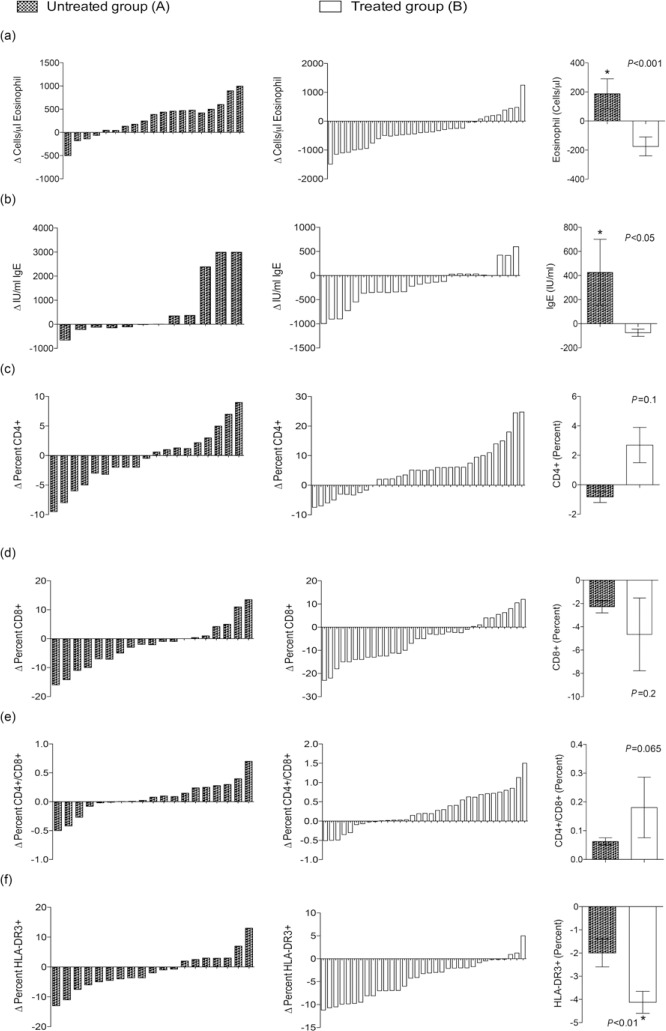
Relative changes (RCs) of the immunological parameters measured in the 2 groups A and B (untreated versus treated) before and after treatment, at baseline (X1) and at 6 to 12 months later (X2) was calculated as follows: RC = [(X_2_-X_1_)/X_1_] x100. For each parameter denoted in lower case alphabets (a to f), the results of the 2 groups (A and B) are presented in parallel. The right panel is the comparison between the cross-sectional mean ± SE of the RCs in the immunological parameters of the 2 groups (A versus B). The *P* value determined by the Mann Whitney rank test is shown. (*P* < 0.05 is statistically significant).

The spectrum and intensity of the parasitic infections, as well as the immune profile, were similar in Groups A (*untreated group*) and B (*treated group*) of the *NEW-Eth-Il* at baseline, as shown in Tables 1 and 3, respectively. However, a detailed analysis of the changes in the immunological profiles at 6 to 12 months (after treatment of Group B) revealed marked differences between these groups. Changes in blood Eosinophils, IgE, CD4, CD8, CD4/CD8 and HLA-DR, are shown in Figure 1(a-f), which clearly demonstrates the marked differences observed in the majority of the individuals in the treated group (B) versus the non-treated group (A). Eradication of the helminth infections in Group B was followed by significant changes in the immune profile, namely, a decrease in the number of eosinophil (*P* = 0.002) and IgE blood levels (*P* = 0.03), an increase in the percentage of CD4+ (*P* = 0.025) and a decrease in the percentage of CD8+ cells (*P* = 0.001), resulting in an increased CD4+/CD8+ ratio (*P* = 0.002), and a decrease in the percentage of activated HLA-DR+CD3+ T-cells (*P* = 0.001) (Table 3).

**Table 3.** Immune parameters (mean±SD) in Group A (in which helminths were not eradicated) and Group B (in which helminths were eradicated) at time 0 and 6 to 12 months later.

Immune Parameter	Group A (n = 19)		Group B (n = 33)			
	0^a^	6-12^b^	p^c^	0^a^	6-12^b^	p^c^
Eosinophils (cells/μl)	905 ± 617	1149 ± 732	0.006	809 ± 558	484 ± 420	0.002
IgE (IU/ml)	2226 ± 1456	2597 ± 1293	NS^d^	1687 ± 1374	1494 ± 1427	0.03
CD4+ (%)	36.7 ± 6.7	36.3 ± 5.8	NS	37.5 ± 7.36	40.3 ± 6.8	0.025
CD4+ (cells/μl)	650 ± 222	697 ± 283	NS	690 ± 193	754 ± 350	NS
CD8+ (%)	36.7 ± 8.4	34.3 ± 8.1	NS	36.1 ± 7.2	30.9 ± 4.2	0.001
CD8+ (cells/μl)	670 ± 287	661 ± 288	NS	702 ± 324	567 ± 188	0.017
CD4+/CD8+ (ratio)	1.06 ± 0.36	1.13 ± 0.43	NS	1.07 ± 0.4	1.32 ± 0.33	0.002
HLA-DR+CD3+ (%)	11.6 ± 9.7	10.26 ± 6.6	NS	9.3 ± 7	5.2 ± 3.8	0.001
CD4+CD45RA+ (%)	21.6 ± 10.9	24.6 ± 9.3	NS	25.8 ± 10.4	23.8 ± 7.2	NS
CD28+CD8+ (%)	40.2 ± 12.1	44 ± 15	NS	42.7 ± 14.6	43.6 ± 12.3	NS

^a^ Blood samples were taken 1 to 3 months after arrival of the *New-Eth-Il* group.

^b^ Blood samples were taken 6 to 12 months after the first blood sample was taken from the *New-Eth-Il* group.

^c^ The statistical difference between each parameter at time 0 and 6 to 12 months was examined by using a paired *t*-test.

^d^ The differences were not statistically significant (NS).

While there were significant difference in the immune parameters of group B, no difference was found in the percentage of naive CD4+CD45RA and CD28+CD8+ cell subsets, and CD4+ count in both groups. In contrast, no significant changes in the immune profile were observed in Group A (Table 3). In fact, we observed a significant increase in eosinophilia in this group (*P* = 0.006). These marked differences can also be seen when the mean relative changes in the immune parameters are compared between the 2 groups (Figure 1 a-f), that is, a statistically significant difference in eosinophil counts, IgE blood levels, and percentage of HLA-DR+CD3+ cells, with non significant differences in the CD4/CD8 ratio (*P* = 0.065). Though individual variation of the immune profile within the groups is evident, the overall result of changes following the treatment are very clear.

### Cytokine secretion by PBMC of helminth infected individuals

**Figure 2.**
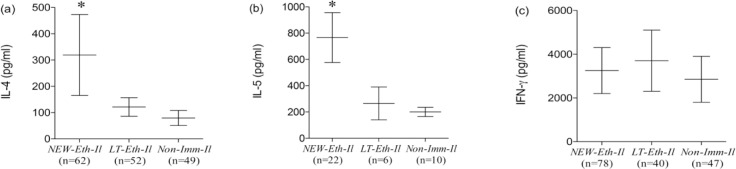
Cytokine Secretion by PHA stimulated PBMC of (a) *NEW-Eth-Il*, (b) *LT-Eth-Il* and (c) *NONImm-Il* groups. The horizontal middle line represents the median (50% of the data is greater than this value). The error bars show the 10th and 90th percentiles of the population (ie, the upper line is the maximum greatest value [MGV] and the lower line is the minimum least value [MLV], excluding the outliers). The number of individuals examined is indicated below each group. The *P* value was determined by the Mann Whitney rank test (*P* < 0.05 is statistically significant).

The levels of IL-4, IL-5 (Th2 type) and IFN-γ (Th1 type) cytokine secretion into supernatants of PHA-stimulated PBMC obtained from *NEW-Eth-Il, LT-Eth-Il* and *NON-Imm-Il* groups are shown in (Figure 2 a to c). Levels of IL-4 and IL-5 secretion, mean±SD were high for *NEW-Eth-Il* participants, (202±154.6 pg/ml and 803.4±349.3 pg/ml, respectively), as compared to *NON-Imm-Il* controls (59.1±60.6 pg/ml and 183.7±109.0 pg/ml, respectively) (*P* < 0.001). The levels of secretion of IL-4 and IL-5 for the *LT-Eth-Il*, were significantly different from those of the *NEW-Eth-Il* (*P* < 0.001) and similar to those of the *NON-Imm-Il* group. The levels of IFN-γ secretion were similar in all 3 groups 2892.2±1492, 3346.0±1869 and 3221.2±1705 pg/ml in the *NEW-Eth-Il, LTEth-Il* and *NON-Imm-Il*, respectively. Longitudinal follow up of IL-4 and IFN-γ secretion before and after antihelminth treatment was available for only 6 individuals. At 6 months after treatment 3/6 individuals still had helminth eggs in their stools. The changes in cytokine secretion after treatment were not significant, however, there was a clear trend toward a reduction in both mean IL-4 and INF-γ levels 6 months after treatment in all 6 individuals, 168.5±89 pg/ml compared to 235.5±214 pg/ml and 3330±2313 pg/ml compared to 4723±1921 pg/ml, respectively.

## DISCUSSION

The purpose of this study was to examine how eradication of chronic helminth infections affects the immune system. This was achieved by comparing the immune profile of recently arrived Ethiopian immigrants to Israel at baseline and 6 to 12 months after treatment of their helminth infections. In order to control for the influence of the changed environment, their immune profiles was compared to that of a parallel group of immigrants, who had similar helminth infections but missed receiving antihelminth treatment (for reasons unrelated to the research). In addition, the immune profile of the *NEW-Eth-Il* group was also compared to that of Ethiopian immigrants living in Israel for at least 5 years and who were free of helminths.

The results of this study confirm our previous observations of marked immune disbalance found in Ethiopian immigrants in Israel [9, 20]. It also confirms similar observations which have been made in Ethiopia [10] and elsewhere in Africa [28, 29]. The change in environment and eradication of helminth infection were associated with decreased immune activation and normalization of the immune profile so that it was similar to that of the non-Ethiopian Israeli population, such as was found in the *LT-Eth-Il* group living in Israel for several years. However, it should be noted, that even after 5 years of living in Israel, the number of CD4+ memory cells, as well as activated CD8+ cells in the *LT-Eth-Il* did not decrease to the same levels of the native Israeli group. Furthermore, the mean eosinophil count and IgE level were still higher than those found in the native Israelis, although these counts were significantly lower than in the *NEW-Eth-Il* group. These findings demonstrate the persistence of long-lasting effects of preexistent chronic helminth infections, in spite of the eradication of parasites and marked environmental changes.

Infection with schistosomes and other helminths is characteristically associated with elevated IgE and eosinophilia, which are both hallmarks of a Th2 cytokine response [30]. Restimulation of PBMC, obtained from schistosome-infected mice and humans, generally leads to secretion of Th2 cytokines [4, 31]. We have previously shown [20] that mitogen stimulation of PBMC induced disparities of cytokine secretion. In the present study we have shown that mitogen induced secretion of both IL-4 and IL-5, Th2 type cytokines, were significantly higher in the *NEW-Eth-Il* compared to the *LT-Eth-Il* group and *NON-Imm-Il* controls. This confirmed our previous reports of a dominant Th2 type profile in helminth infected individuals [20, 32]. However, the mitogen induced secretion of the Th1- type cytokine, IFN-γ, was similar in the *NEW-Eth-Il* and *LT-Eth-Il* groups and *NON-Imm-Il* controls, contrary to our previous finding [20]. The difference in the results for IFN-γ secretion may be because of the high individual variations and the larger number of samples in the previous study. Dynamic fluctuations in type 1 or type 2 cytokine responses have been observed in murine models of schistosomiasis [33]. These studies showed that the shift from a Th1 dominant to Th2-dominant response coincides with egg production [34]. In humans, elevated IFN-γ antigen specific responses have been shown to be associated with acute schistosomiasis as well as with hepatosplenic disease [6, 35]. In addition IL-5 responses have also been observed in patients with acute schistosomiasis, which suggests the presence of a mixed type 1 and type 2 cytokine profile [32]. During the chronic stage of infection, type 2 responses are diminished and there is some increase in type 1 responses.

The long-term effect of antihelminth treatment on cytokine secretion can be inferred from the results obtained in the *LT-Eth-Il* group. These individuals had been uninfected by helminths for some years and their mitogen-induced IL-4 and IL-5 secretion by PBMC decreased to near-normal levels, similar to those of the *NON-Imm-Il* controls. However, the effects of environmental factors on cytokine secretion, due to living in Israel for several years, cannot be ruled out. The kinetics of changes in cytokine secretion, before and 6 months after treatment was studied in a small group of people of whom 50% were still infected with helminths after treatment. However, the relatively high mean IFN-γ level secretion seen in this specific group on arrival in Israel decreased significantly 6 months after treatment and IL-4 levels clearly decreased in those who were uninfected with helminths but was still significantly higher than the control level. Grogan *et al*, [11] have shown that 5 weeks of antihelminth treatment for chronic infection with *Schistosoma haematobium* resulted in a significant decrease in worm load, an increase in proliferative responses to adult worm antigen and an increased secretion of specific IL-4 with no change in IL-5. Thus, chronic infection was associated with a reversible down regulation of specific IL-4 release in contrast to the reversible increased IL-4 secretion by mitogenic stimulation, which we observed.

Helminth infection affects the immune responses to inciting antigens and pathogens and decreases the ability of the host to generate a protective immunity to both HIV and mycobacteria [16]. Thus, these chronic infections could have a major impact on the host's immune system and on the susceptibility and spread of HIV in the developing world. We have earlier shown that plasma viral load in HIV asymptomatic and antiretroviral naive patients in Ethiopia was highly correlated with the number of helminth eggs (mainly *Ascaris lumbricoides* and *Trichuris trichiura*) in the stool [15], and it has been suggested that successful deworming is associated with a significant decrease in HIV plasma viral load and CD4+ counts [36, 37]. Studies on HIV-1 helminth co-infection have also shown no decrease in viral load [38, 39]. The discrepancies in the results may be attributable to the differences of the helminth species, parasite infection load, the length of the follow-up period, frequency of deworming and plasma concentrations of HIV-1 RNA at base line. Our study has shown that the immunological recovery from Schistosomiasis is longer than that of soil-transmitted helminth infection. Underlying helminth infections may also jeopardize the efficacy of candidate vaccines to develop a protective immunity to HIV and tuberculosis [23, 24].

## CONCLUSION

In conclusion, the primary outcome of the treatment group was clearing the helminth infection and reducing the parasitic load; beyond this, the study showed that treatment of soil-transmitted helminthiasis and schistosomiasis had a significant effect on IgE levels and eosinophil counts, CD4/CD8 ratio, proportion of HLA-DR+CD3+, HLA-DR+CD4+ and HLA-DR+CD8+ cells, CD45RA+CD4+ (naive) and CD28+CD8+ cells, CD45RO+CD4+ (memory) cells, and secretion of IL-4 and IL-5. Thus, deworming which normalizes the immune system and restores immune responses is feasible on a large scale and is relatively inexpensive. We recommend that this procedure should be speedily carried out in developing countries.

## LIMITATIONS OF THE STUDY

The small sample size of each group and the lack of socio-demographic and anthropometric data are considered as the limitations of this study.
